# A first phylogenetic analysis reveals a new arboreal tarantula genus from South America with description of a new species and two new species of *Tapinauchenius* Ausserer, 1871 (Araneae, Mygalomorphae, Theraphosidae)

**DOI:** 10.3897/zookeys.784.26521

**Published:** 2018-09-12

**Authors:** Martin Hüsser

**Affiliations:** 1 Rüteneneweg 3, 8964 Rudolfstetten, Switzerland Unaffiliated Rudolfstetten Switzerland

**Keywords:** arboreal, morphology, tarantula, phylogenomics, Psalmopoeinae

## Abstract

Based on molecular and morphological phylogenetic analyses a new genus of Theraphosidae is described, *Pseudoclamoris***gen. n.***Tapinaucheniusgigas* and *Tapinaucheniuselenae* are transferred to *Pseudoclamoris* and a new species of *Pseudoclamoris* from the Amazon Region is described: *P.burgessi***sp. n.** Two new species of *Tapinauchenius* from the Caribbean are described: *T.rasti***sp. n.** and *T.polybotes***sp. n.***Tapinaucheniussubcaeruleus* is considered a *nomen dubium*. Psalmopoeinae subfamily is diagnosed based on molecular and morphological phylogenies, and *Pseudoclamoris***gen. n.** and *Ephebopus* Simon, 1892 are included. A taxonomic key for Psalmopoeinae genera *Tapinauchenius*, *Pseudoclamoris*, *Psalmopoeus*, and *Ephebopus* is provided.

## Introduction

The Theraphosidae are among the largest Mygalomorphae and along with the Theraphosinae, members of the Aviculariinae remain of great interest to arachnologists due to their unresolved phylogenetic relationship, exhibiting extreme diversity all over south America.

The genera *Tapinauchenius* Ausserer, 1871 and *Psalmopoeus* Pocock, 1895 have never been reviewed or revised before, even though new species have been described in recent years (Mendoza 2014). Ausserer (1871) defined the genus *Tapinauchenius* for the already-described species *Mygaleplumipes* C. L. Koch in 1842, based on the absence of stridulatory organ. The newly designated type species, *Tapinaucheniusplumipes*, was only known from a mature male until Becker (1879) described the female of the species as *Aviculariadeborri*, which later got referred as *T.plumipes* by Schmidt (1994a), based on morphological similarities and same type locality of Paramaribo, Suriname. Simon (1892) placed *Mygalesanctivincentii* Walckenaer, 1837 in *Tapinauchenius*: *T.sanctivincentii* is only known from a single female specimen, of which the type is apparently lost (Rollard, pers. comm.). *Tapinaucheniuslatipes* was described by L. Koch in 1875, based on a single male specimen from Venezuela. However, this species is supposed to differ from *T.plumipes* by its ocular alignment, but Koch did not specify in which way these differ exactly and no further diagnosis was provided. The female of *T.latipes* was subsequently described by Schiapelli and Gerschman (1945). Two years later, Caporiacco (1947) described *Tapinaucheniusconcolor* from Guyana, but erroneously placed it in *Pachistopelma*. This placement was found to be incorrect by Bertani (2012), who transferred the species to *Tapinauchenius*, referring to its type specimen as an immature male. Caporiacco (1954) described *Tapinaucheniusgigas* from French Guyana based on a female specimen, while Schmidt (1994b) described the male of this species. In the following years, Schmidt worked extensively on the taxonomy of Theraphosidae and described a number of additional species of *Tapinauchenius*, namely *Tapinaucheniuselenae* Schmidt, 1994 from Ecuador, *Tapinaucheniusbrunneus* Schmidt, 1995 from the Amazon region of Mato Grosso, Brazil, based on a single male specimen and *Tapinaucheniuscupreus* Schmidt & Bauer, 1996 from Ecuador. Schmidt (1995b) also described *Tapinaucheniuspurpureus* from French Guyana from both sexes, but the species was later found to be a junior synonym of *Tapinaucheniusviolaceus* (Mello-Leitão, 1930) by West et al. (2008). Bauer and Antonelli (1997) described *Tapinaucheniussubcaeruleus* from Ecuador, based on material from pet trade lacking further information on the exact collection locality. Auer et al. (2007) presented a summary of all known *Tapinauchenius* available in the pet trade and provided valuable comments and suggestions regarding the groupings in the genus. For the first time, two groups within *Tapinauchenius* were recognized, based on the colouration of juvenile specimens. These groups were the *gigas* group, with ontogenetic pattern change, comprising *T.gigas*, *T.elenae* and *T.subcaeruleus*, and the *plumipes* group, without ontogenetic pattern change, consisting of *T.plumipes*, *T.latipes*, *T.violaceus*, *T.sanctivincenti*, and *T.cupreus*. West et al. (2008) showed images of lyrate setae on prolateral palpal coxa in an unidentified *Tapinauchenius* species from Peru but did not provide further comments besides the SEM images of the mentioned structure. Bertani (2012) included *Tapinauchenius* and *Psalmopoeus* in his cladistic analysis and placed them within Aviculariinae while describing new genera and species in the same subfamily.

The latest changes to the supposed sister taxon of *Tapinauchenius*, *Psalmopoeus*, were made by Mendoza (2014), who described *Psalmopoeusvictori* from Mexico, providing some insight on the northernmost distribution of the Psalmopoeinae clade. Mendoza (2014) followed Bertani (2012) by placing *Psalmopoeus*, *Tapinauchenius*, and *Ephebopus* Simon, 1892 in the Aviculariinae. In the most recent study on Aviculariinae, Fukushima and Bertani (2017) used an in-depth morphological cladistic analysis to justify the placement of *Psalmopoeus*, *Tapinauchenius*, and *Ephebopus* in Aviculariinae. Contrary to their results, the proposals of Samm and Schmidt (2010), Lüddecke et al. (2018) and Turner et al. (2018) are followed here by placing *Psalmopoeus*, *Tapinauchenius*, as well as *Pseudoclamoris* gen. n. in Psalmopoeinae. Molecular based data from Lüddecke et al. (2018), Turner et al. (2018), and the work presented here strongly suggest a close phylogenetic relationship between Psalmopoeinae and Schismatothelinae, while morphological data further revealed that these groups possibly share several synapomorphies.

During the process of revising *Tapinauchenius* and *Psalmopoeus*, significant morphological traits in certain species of the genus *Tapinauchenius* were revealed (see Figs 2, 13). These features have been studied and were included in the morphological cladistic analysis. In addition, tissue samples were sequenced for molecular phylogenetic analyses. Combined analyses lead to the establishment of a new genus to accommodate two *Tapinauchenius* species: *Pseudoclamoris* gen. n., comprising *Pseudoclamorisgigas* comb. n., *Pseudoclamoriselenae* comb. n., and *Pseudoclamorisburgessi* sp. n. Morphological and genetic material from two Caribbean Islands lead to the description of two new species of *Tapinauchenius*: *Tapinaucheniusrasti* sp. n. from Union Island, Lesser Antilles and *Tapinaucheniuspolybotes* sp. n. from the island of Saint Lucia, Lesser Antilles.

## Materials and methods

Measurements were taken with an ocular micrometre of a Nikon SMZ645 binocular microscope. Measurements were made along the central axis of the measured structures and are given in millimetres. Measurements of leg and palpal segments were taken dorsally. The eye measurements were taken from the widest spans of the lens, AME in dorsal view, ALE, PLE, and PME in dorsolateral view. Measurement of the total body length, including cephalothorax and abdomen without spinnerets, were made using a digital caliper. As measurements of total body length include non-sclerotized tissue of the abdomen, they should be considered to be approximates only.

Appendage measurements were based on right appendages (unless otherwise stated); palpal tibia & leg I – retrolateral, legs III and IV – prolateral, extent of metatarsal scopulation – ventral. The lengths of the leg articles were taken from the mid-proximal point of articulation to the mid-distal point of the article (sensu Coyle 1995, Bond et al. 2012, Bond and Godwin 2013, and Hamilton et al. 2016).

Genitalia were prepared according to von Wirth (2006) and photographed with a Canon EOS 6D DSLR camera body using the Macro Photo MP-E 65mm f/2.8 1–5× Manual Focus Lens for EOS mounted on a Cognisys Automatic Stacking Rail. Thirty (3×) photographs were taken of each specimen in ethanol under glass and stacked using Zerene Stacking Software. The scientific picture plate layout follows Fukushima and Bertani (2018).

The extent of the tarsal and metatarsal scopulae on the ventral side of both leg segments was expressed as a percentage of the total length of the segment from the apical end. Terminology used to describe the male palpal bulb structures follows Bertani (2000). Maps were made with SimpleMappr, an online tool to produce species distribution maps (Shorthouse 2010).

### Molecular techniques and phylogenomic analyses

An important aspect of this work involves the genetic studies carried out. The following primers used for the polymerase chain reactions (PCRs) were used in this paper. The primers LCO-1490 (5’ GGT CAA CAA ATC ATA AAG ATA TTG G 3’) and HCO-2198 (5’ TAA ACT TCA GGG TGA CCA AAA AAT CA 3’) (Folmer et al. 1994) target a 710-base pairs fragment of the COI mitochondrial region. The primer pair 16SAL-Tarantula-F1 (5’ GTG CTA AGG TAG CAY AAT 3’) and 16S-Tarantula-R2 (5’ TAA TTC AAC ATC GAG GTC 3’) (Lüddecke et al. 2018) amplify a fragment of about 270 base pairs of the mitochondrial 16S rRNA gene. Finally, the primers 28SO F (5’ TCG GAA GGA ACC AGC TAC TA 3’) and 28SC R (5’ GAA ACT GCT CAA AGG TAA ACG G 3’) (Hedin and Maddison 2001) were used to amplify a fragment of 760–800 base pairs of the nuclear 28S rRNA gene.

A negative control that contained no DNA was included in every PCR round to check for cross-contamination. PCR products were run on agarose gels and imaged under UV light to verify the amplicon size. The PCR products were bi-directionally sequenced using the PCR primers. Electropherogram analysis and overlapping was conducted using Geneious 8.1.8. During the electropherogram analysis, the primer annealing regions and the low-quality regions at both ends of each electropherogram were trimmed (error probability limit of 0.03).

Specimens used in this first phylogenetic approach of this subfamily are listed in Supplementary Data, originating partly from pet trade and wild caught specimens. All

specimens sampled by use of non-lethal techniques (leg autotomy) appeared to undergo very little stress. All specimens survived the respective procedures (until preserved for vouchers). All data (molecular, morphological, etc.) used to establish these species hypotheses have been deposited in the Dryad Data Repository (doi: 10.5061/dryad.k6483cr). GenBank data for Theraphosidae outgroup has been used, namely *Selenocosmiajavanensis* (MG273512.1), *Theraphosaapophysis* (KY017414.1) and *Poecilotheriametallica* (KY016161.1).

Maximum likelihood analyses were conducted using RAxML version 8.2 with the GTRGAMMA model and 1000 rapid bootstrap replicates. Bayesian analyses were conducted with MrBayes 3.2.6 on the CIPRES portal (Miller et al. 2010). Analyses were run for 10 million generations, logging every 1000 generations. Analyses were carried out on the authors’ computers for crosscheck reference in order to prevent any wrong settings for calculation. Figure 16 shows the preferred tree which is used for discussion.

Specimens from the Forschungsinstitut und Naturmuseum Senckenberg, Frankfurt (**SMF**), Museum für Naturkunde der Humboldt-Universität, Berlin **(ZMB**), Museum National d’Histoire Naturelle, Paris (**MNHN–AR**), and the personal collection of the author (**MHCOL**) were examined as part of this work. The general description format follows Hamilton et al. (2016), with modifications, mainly of setae and trichobothria patterns, which were not studied in detail here. The description format of Hamilton et al. (2016) was used to evaluate morphological variation when more material was available.

Abbreviations (see Hamilton et al. 2016: fig. 3):

**Cl** length of the carapace;

**Cw** width of the carapace;

**LBl** labial length;

**LBw** labial width;

**F1** femur I length (retrolateral aspect);

**F1w** femur I width;

**P1** patella I length;

**T1** tibia I length;

**M1** metatarsus I length;

**A1** tarsus I length;

**F3** femur III length (prolateral aspect);

**F3w** femur III width;

**P3** patella III length;

**T3** tibia III length;

**M3** metatarsus III length;

**A3** tarsus III length;

**F4** femur IV length (prolateral aspect);

**F4w** femur IV width;

**P4** patella IV length;

**T4** tibia IV length;

**M4** metatarsus IV length;

**A4** tarsus IV length;

**PTl** palpal tibia length (retrolateral aspect);

**PTw** palpal tibia width;

**SC3** ratio of the extent of metatarsus III scopulation (length of scopulation/ventral length of metatarsus III);

**SC4** ratio of the extent of metatarsus IV scopulation (length of scopulation/ventral length of metatarsus IV);

**AER** Anterior Eye Row;

**PER** Posterior Eye Row;

**MPT** most parsimonious tree;

**CI/ci** consistency index;

**RI/ri** retention index;

**hi** homoplasy index;

**G-fit** Goloboff fits.

Material examined (for detailed information to examined and sequenced material, see Suppl. material 1): Male holotype and female paratype of *Tapinaucheniuselenae* from Tena, Ecuador, SMF (37349), leg. Hirsch, VIII, 1992; examined. Female lectotype of *Tapinaucheniusgigas* from French Guyana, MNHN (AR14637), leg. Sammler; and male from French Guyana, SMF (38050), leg. Verdez; examined. Male holotype (38042) and female paratype (38046) of *Tapinaucheniuscupreus* from Ecuador, SMF, leg. Bullmer; examined. Female holotype and male paratype of *Psalmopoeuslangenbucheri* from Caripe, Venezuela, SMF (58086), leg. Langenbucher; examined. Male holotype of *Tapinaucheniusbrunneus* from Mato Grosso, Brazil, SMF (38008), leg. Ockert; examined. 2 male syntypes (legs sep.), 1 male bulb (sep.) of *Tapinaucheniusplumipes* from Suriname, ZMB (MYR 2044); examined. Female holotype (38042) and male paratype (38046) of *Tapinaucheniusviolaceus* from French Guyana, SMF, leg. Braunshausen, 1994; examined.

Other material examined: *Tapinaucheniuselenae*: female exuviae (SMF 57952). *Tapinaucheniusgigas*: 1 female (SMF 38050), 3 females (MHCOL_00131, 00112. 00121) and 4 males (MHCOL_00201, 00161, 00174, 00189). *Tapinaucheniuscupreus*: female exuviae (SMF 57925, SMF 58281), 3 females (MHCOL_0012, 0097, 027) and 2 males (MHCOL_0289, 0301). *Psalmopoeuslangenbucheri*: 3 females (MHCOL_00055, 00198, 00401) and 3 males (MHCOL_00501, 00512, 00518). *Tapinaucheniusplumipes*: 3 females (MHCOL_0192, 0211, 0312) and 4 males (MHCOL_0089, 0092, 0212, 0415). *Tapinaucheniusviolaceus*: 3 females (MHCOL_0152, 0243, 0442) and 2 males (MHCOL_0163, 0288).

### Phylogenetics

Members of the family Theraphosidae are known for their morphological homogeneity (see Raven 1985, Goloboff 1993, Bertani 2001, Bond et al. 2012). Considering this, it is extremely difficult to find characteristics that offer the required level of stability and uniqueness to be useful for analyses. For example, similarly to Fukushima and Bertani (2017), certain ratios between the extremities (such as legs and palps) and the carapace itself were set out to apply. However, these were found not suitable for further analyses.

As shown by Fukushima and Bertani (2017), a revision based solely on morphological characters is extremely difficult, as the variation within characters is often high. It has been shown that an approach that combines classical morphological taxonomy with complementary techniques, such as DNA sequencing, is more accurate and reliable due to the larger amount of data to work with (Bond et al. 2012; Hamilton et al. 2016).

### Morphological analysis

A data matrix consisting of 19 taxa and 30 unordered, parsimony informative characters (Тable 1) was analyzed in PAUP* 4.0a162 Swofford (2018) under the maximum parsimony criterium at 10 replicates with random addition sequence using tree bisection reconnection (TBR). Bootstrap support was evaluated at 1000 replicates after successive reweighting according to the mean ci at a base-weight of 10. Table 1 and 2 together with Figure 17 are displaying the results of this work.

**Table 1:** Character step matrix used for cladistic analysis.

Taxa										1										2										3
	1	2	3	4	5	6	7	8	9	0	1	2	3	4	5	6	7	8	9	0	1	2	3	4	5	6	7	8	9	0
* Stenoterommata *	0	0	–	0	0	0	0	0	0	0	0	0	?	0	0	0	0	0	0	0	0	–	0	0	0	0	0	0	0	0
* Sason *	0	2	–	0	–	0	0	1	0	0	0	0	?	0	0	0	0	0	1	1	0	0	0	0	0	0	0	0	0	0
* Antillena *	1	4	–	1	0	0	1	1	0	0	0	0	0	1	1	1	1	1	3	0	0	1	0	3	1	0	1	1	0	1
* Avicularia *	1	3	–	1	0	0	1	0	0	0	0	0	0	1	1	1	1	1	3	0	0	1	1	2	0	1	1	2	0	1
* Caribena *	1	4	–	1	0	0	1	1	0	0	0	0	0	1	1	1	1	1	3	0	0	1	1	3	1	0	0	1	0	1
* Pachistopelma *	1	3	–	1	0	0	1	0	0	0	0	0	0	1	1	1	1	1	3	0	0	1	1	2	1	0	0	2	0	1
* Ephebopus *	1	1	1	2	1	1	1	0	0	0	0	0	1	1	1	1	1	0	3	0	1	1	1	0	0	1	1	0	1	0
* Holothele *	1	1	1	0	0	1	1	0	0	1	1	1	1	1	0	0	1	0	2	1	1	0	0	2	0	0	0	0	0	0
* Schismatothele *	1	1	0	0	0	1	1	0	0	1	1	1	1	1	0	0	1	0	2	1	1	0	0	4	0	0	0	0	0	0
* P. gigas *	1	1	0	0	1	1	1	0	1	0	0	0	1	1	1	1	1	1	3	0	1	1	1	2	0	1	1	0	0	0
* P. elenae *	1	1	0	0	1	1	1	0	1	0	0	0	1	1	1	1	1	1	3	0	1	1	1	2	0	1	1	0	0	0
* P. burgessi *	1	1	1	0	1	1	1	0	1	0	0	0	1	1	1	1	1	1	3	0	1	1	1	2	0	1	1	0	0	0
* P. irminia *	1	1	0	0	1	1	1	0	2	0	0	0	1	1	1	1	1	1	3	0	1	1	0	1	0	0	0	0	0	0
* P. reduncus *	1	1	0	0	1	1	1	0	2	0	0	0	1	1	1	1	1	1	3	0	1	1	1	0	0	0	0	0	0	0
* P. cambridgei *	1	1	0	0	1	1	1	0	2	0	0	0	1	1	1	1	1	1	3	0	1	1	0	1	0	0	0	0	0	0
* T. plumipes *	1	1	1	0	1	1	1	0	0	0	0	0	1	1	1	1	1	0	3	0	1	1	0	4	0	0	1	0	0	0
* T. rasti *	1	1	2	0	1	1	1	0	0	0	0	0	1	1	1	1	1	0	3	0	1	1	0	4	0	0	1	0	0	0
* T. polybotes *	1	1	1	0	1	1	1	0	0	0	0	0	1	1	1	1	1	0	3	0	1	1	0	4	0	0	1	0	1	0

## Characters

Characters from Guadanucci (2014) (2, 3, 5, 8, 9, 10, 11, 12, 13, 15, 15, 18, 19, 20, 21, 22, 23) and Fukushima and Bertani (2017) (1, 6, 7, 14, 16, 17, 24, 25, 26, 27, 28 29,30) were combined and slightly modified (2, 3, 9, 23, 29). Character 4 was added in order to account for the autapomorphy (presence of urticating setae on palpal femora) of *Ephebopus*, alongside the presence of abdominal urticating setae in Aviculariinae. Abdominal urticating setae have also been acquired in Theraphosinae, whereas the state scored for *Ephebopus* is unique among Theraphosidae:

(1) Cymbial lobes: (0) distinct from each other; (1) similar to each other.

(2) Male tibial apophysis: (0) absent; (1) present, prolateral branch well developed with spine, Guadanucci et al. (2007: figs. 10 and 11); (2) present, weakly developed, with megaspine, Guadanucci (2014: fig. 16); (3) present, composed of spinifom setae, von Wirth and Striffler (2005: fig. 26); (4) present, prolateral branch well developed and fused with retrolateral branch; Bertani (2001: figs. 22 – 23).

(3) Male tibial spur: number of spines on Rap: (0) 1 short and strong spine on the inner face and 1 short and strong spine on top; (1) 1 short and strong spine on the inner face and 2 short and strong spines on outer face; (2) 1 short and strong spine on the inner face and 1 short and 3 strong spines on outer face (Figure 11c).

(4) Urticating setae: (0) absent; (1) present on opisthosoma; (2) present on palpal femur.

(5) Multilobular spermathecae: (0) absent; (1) present, Guadanucci (2007: Figure 6).

(6) Spermathecae cuticula sclerotization: (0) slightly (thin and soft); (1) strongly sclerotized.

(7) Anterior maxillary lobe (anterior ventral corner, Raven 1994): (0) not produced; (1) produced.

(8) Number of maxillary cuspules: (0) several (more than 30); (1) few (less than 15).

(9) Lyra on prolateral maxilla. 1/4^th^ of the surface: (0) absent; (1) field of needle-like setae (Figure 2); (2) curved lyriform setae (Figure 3).

(10) Labial cuspules located: (0) on a flat area; (1) on a raised area.

(11) Labial cuspules density: (0) weakly dense, spread over the labium; (1) strongly dense, concentrated at the apical portion (approximately 200 cuspules).

(12) Sternal anterior bulge: (0) absent; (1) present.

(13) Armed Tarsal Claw: (0) absent; (1) present.

(14) Third tarsal claw: (0) absent; (1) present.

(15) Leg spines: (0) present on tibiae and metatarsi; (1) absent on tibiae and metatarsi.

(16) Tarsal and metatarsal scopula laterally projected: (0) absent; (1) present.

(17) Tarsal scopula I: (0) divided, Guadanucci (2011a: fig. 2); (1) undivided.

(18) Tarsal scopula IV: (0) divided; (1) undivided.

(19) Tarsal trichobothria disposition: (0) absent; (1) small compact group, Guadanucci (2012: fig. 1); (2) two rows, Guadanucci (2012: fig. 47); (3) U-shaped, Guadanucci (2012: fig. 39).

(20) Tibial thickened trichobothria: (0) absent; (1) present.

(21) Tibial clavate trichobothria: (0) absent¨; (1) present.

(22) Tibial clavate trichobothria disposition: (0) straight row, Guadanucci (2012: fig. 189a); (1) compact group, Guadanucci (2012: fig. 161).

(23) Ontogenetic change of colour pattern: (0) pattern remains practically the same; (1) pattern presents drastic changes.

(24) Dorsal abdominal pattern in juveniles: (0) homogeneous; (1) herringbone; (2) central longitudinal black stripe with 5–6 lateral stripes, connecting or not with the central stripe; (3) central longitudinal reddish stripe inside a dark area with zigzag border; (4) longitudinal central stripe of a different colour of remaining abdomen.

(25) Body colouration in juveniles: (0) matte/dull (brown or grey); (1) iridescent (green or blue).

(26) Colouration of tarsi in juveniles: (0) same colour of other articles; (1) black.

(27) Distribution of abdominal setae in females: (0) homogeneous; (1) heterogeneous, with long guard-setae grouped on lateral and dorso anterior areas.

(28) Proximal part of embolus in frontal view, shape: (0) straight; (1) slightly curved; (2) strongly curved.

(29) Proximal part of embolus in frontal view, state: (0) no curvature; (1) strongly S-shaped curvature, West et al. (2008: Figs 7–9).

(30) Anterior eye row: (0) straight; (1) procurved.

## Results

Morphology based searches in PAUP* resulted in a single most parsimonious trees (length = 58, Ci = 0.7241, Ri = 0.8298; Figure 17; character changes see Table 2, character diagnostics see Table 3). Within Theraphosidae, Psalmopoeinae and Aviculariinae share a number of synapomorphies in characters 5 (Spermathecal receptacles), 15 (leg spines absent on tibiae and metatarsi), 16 (tarsal and metatarsal scopula laterally projected) and 22 (tibial clavate trichobothria disposition), resulting in a good support for this grouping (100). *Psalmopoeus* is found monophyletic and well-supported (99) based on synapomorphies in characters 9 (lyra on prolateral maxilla; see West et al. 2008) and 18 (tarsal scopula IV), the latter with a parallelism in *Pseudoclamoris*+Aviculariinae. *Psalmopoeuscambridgei* and *Psalmopoeusirminia* differ mainly by their colouration and there is convincing evidence (97) these two are sister taxa, as their juveniles share the same state in character 24 (dorsal abdominal pattern in juveniles). Character 24 was found to introduce homoplasy, but not because the unique state found in these two species of *Psalmopoeus*, but due to ambiguous changes in Schismatothelinae. The character should be further tested in this group to account for its stability correctly (in prep.). *Psalmopoeusreduncus* differs from the other species by its apomorphy in character 23 (ontogenetic change of colour pattern), with a parallelism in *Ephebopus+Pseudoclamoris*+Aviculariinae and a reversal in *Antillena*. *Tapinauchenius* and other Psalmopoeinae+Aviculariinae share a single synapomorphy in character 27 (distribution of abdominal setae in females), but as this character seems to be rather homoplastic (ci = 0.333), support for this grouping was rather low (61), as to be expected. However, the state in character 3 (male tibial apophysis: number of spines on Rap), with a parallelism in *Holothele* Karsch, 1879, might be another synapomorphy to support this grouping. *Tapinauchenius* is found monophyletic based on character 24 (dorsal abdominal pattern in juveniles), but as the same state can also be found in *Schismatothele*, this monophyly is not well supported (74). *T.rasti* sp. n. males differ from those of the other *Tapinauchenius* species by the number of spines on Rap based on character 3, whereas *T.polybotes* sp. n. has a distinct curvature of its embolus, character 29, otherwise only found in *Ephebopus* (ci = 0.5). *Ephebopus*, *Pseudoclamoris* gen. n. and the Aviculariinae share synapomorphies in characters 23 (ontogenetic change of colour pattern) and 26 (colouration of tarsi in juveniles), a grouping that is not well supported (62), due to both of these characters exhibiting homoplasy. Urticating setae on palpal femora are found to be an autapomorphy of *Ephebopus*, whereas abdominal urticating setae are a synapomorphy of Aviculariinae with a possible parallelism in Theraphosinae. *Pseudoclamoris* and Aviculariinae share the same states in characters 18 (tarsal scopula IV) and 24 (dorsal abdominal pattern in juveniles). As both of these characters introduce homoplasy, this is found another of the not so well supported groupings (75). *Pseudoclamoris* is found monophyletic and forms a more stable grouping (85) based on their unique state in character 9 (lyra on prolateral maxilla). The possibility of the structure found in *Pseudoclamoris* being an earlier stage of a lyra found in *Psalmopoeus* was tested (ctype ord: 9) and resulted in four trees (length = 59, Ci = 0.7119, Ri = 0.8247), each one step longer than the presented topology resulting in a less resolved strict consensus, while the presented topology was still found part of the treespace. A lyra can also be found in Dipluridae Simon, 1889, *Idiommata* Ausserer, 1871 and Selenocosmiinae Simon, 1889 (Raven 1985). The lyra in Selenocosmiinae might become more complex, but is possibly secondarily lost in parts of this subfamily. While certainly being homoplastic in larger context, the development of such a lyra in Selenocosmiinae might shed some light on how to evaluate this character in Psalmopoeinae. *P.gigas* and *P.elenae* are found sister taxa based on the number of spines on Rap and form a rather stable grouping (76). Developments in Aviculariinae resemble those presented by Fukushima and Bertani (2017); also see Table 2.

Spermathecae, male palpal bulb, and tibial apophysis shape as well as somatic characters (except colouration traits) of *Psalmopoeus*, *Tapinauchenius* and *Pseudoclamoris* gen. n. are very similar among different populations and species. Therefore, as occurring in *Aphonopelma* (see Hamilton et al. 2016) and *Avicularia* (see Fukushima and Bertani 2017) it is very probable that we can only access the real diversity of this clade using multiple approaches for an accurate definition of specific and generic boundaries.

Morphologically cryptic species are an increasingly recurrent problem on traditional zoological taxonomy (Satler et al. 2013). Boundaries of many Psalmopoeinae species could not be delimited using the current morphological tools and data here.

**Table 2:** Character changes connected to nodes in preferred topology resulting from cladistic analysis; ambiguous changes in italics.

Node	Character change(s)
33 > *Stenoterommata*	*2 (1<->0)*, 19 (0<->1), 20, (0<->1)
33 > *Sason*	2 (1>2), 8 (0>1)
33 > 32	1 (0>1), 6 (0>1), 7 (0>1), 14 (1>0), 17 (0>1), *19 (1>2)*, 21 (0>1)
32 > 30	5 (0>1), 15 (0>1), 16 (0>1), *19 (2>3)*, *20 (1>0)*, 22 (0>1)
30 > 27	*3 (0>1)*, 27 (0>1)
27 > 25	23 (0>1), 26 (1>0)
25 > *24*	18 (0>1), 24 (0>2)
24 > *21*	2 (1>3), 4 (0>1), 5 (1>0), 6 (1>0), 13 (1>0), 21 (1>0), 28 (0>2), 30 (0>1)
21 > 20	25 (0>1), 26 (1>0), *27 (1>0)*
20 > 19	2 (3>4), 8 (0>1), 24 (2>3), 28 (2>1)
19 > *Antillena*	23 (1>0), *27 (0>1)*
24 > 23	9 (0>1)
25 > *Ephebopus*	4 (0>2), 29 (0>1)
27 > 26	24 (0>4)
26 > *T.rasti* sp. n.	3 (1>2)
26 > *T.polybotes* sp. n.	29 (0>1)
30 > 29	9 (0>2), 18 (0>1)
29 > 28	24 (0>1)
29 > *P.reduncus*	23 (0>1)
32 > 31	10 (0>1), 11 (0>1), 12 (0>1), *24 (0>2)*
31 > *Holothele*	*3 (0>1)*
30 > *Schismatothele*	*24 (2>4)*

**Table 3.** Character diagnostics for preferred topology resulting from cladistic analysis.

Character	Ci	Ri	G-fit
1	1.000	1.000	1.000
2	1.000	1.000	1.000
3	0.500	0.500	0.600
4	1.000	1.000	1.000
5	0.500	0.833	0.750
6	0.500	0.800	0.750
7	1.000	1.000	1.000
8	0.500	0.500	0.750
9	1.000	1.000	1.000
10	1.000	1.000	1.000
11	1.000	1.000	1.000
12	1.000	1.000	1.000
13	1.000	1.000	1.000
14	1.000	1.000	1.000
15	1.000	1.000	1.000
16	1.000	1.000	1.000
17	1.000	1.000	1.000
18	0.500	0.857	0.750
19	1.000	1.000	1.000
20	0.500	0.500	0.750
21	0.500	0.800	0.750
22	1.000	1.000	1.000
23	0.333	0.714	0.600
24	0.667	0.750	0.600
25	1.000	1.000	1.000
26	0.500	0.750	0.750
27	0.333	0.750	0.600
28	1.000	1.000	1.000
29	0.500	0.000	0.750
30	1.000	1.000	1.000

### Molecular results

In general, the combined gene tree has a strong support in both BA and ML (100/100) for the subfamily of Psalmopoeinae and shows two distinct lineages within Schismatothelinae.

*Tapinaucheniuspolybotes* sp. n. displays as a sister species to *T.cupreus* even though their geographic locations, based upon their type locality, are hundreds of miles apart. *Tapinaucheniusrasti* sp. n., which is geographically next to *T.polybotes* sp. n. and *T.plumipes*, shows as a distinct lineage within the grouping of *Tapinauchenius* forming a strong support for the genus (100/100)

The genus *Ephebopus* shows close relationship to *Tapinauchenius* in BA (97) but not in ML (40), making the placement of it rather difficult and unresolved at this stage. Further sampling of this genus is needed to clarify the correct placement regarding their relationship within the subfamily Psalmopoeinae.

*Pseudoclamoris* gen. n. forms a unique clade within the Psalmopoeinae subfamily with strong support of 100/97. At this stage it is unclear whether *Pseudoclamoris* gen. n. is the sister genus of *Tapinauchenius* or *Psalmopoeus*, as it’s not possible to determine the evolutionary progress of the stridulatory organ. The presence of needle-like field of setae on the proximal maxilla, described here, is unique within Psalmopoeinae and clearly differentiates this genus from other genera. Phylogenomic analysis also show no close relationship to Selenocosmiinae subfamily, therefore the scoring of stridulatory lyra in morphology cladistic is questionable in general.

Based on our analysis, *Ephebopus* appears to be the sister group to *Tapinauchenius* as also shown by West et al. (2008). Therefore, the definition of Psalmopoeinae must be altered to accommodate *Pseudoclamoris* gen. n. and *Ephebopus*, as it fits the result of the morphologically based cladistic analysis by West et al. (2008), as well as the two approaches presented here.

Even though *Psalmopoeus* is not in focus in this work, analysis of several species of *Psalmopoeus* might lead to a possible paraphyly of the genus. *P.cambridgei* and *P.irminia* show distinct morphological characteristics (curvature of stridulatory organ, Mendoza (2014b: Figs 16 and 17) and juvenile colouration and ontogenetic pattern change differs from other species of the genus) which supports the hypothesis of an independent evolutionary lineage. The type species of *Psalmopoeus*, *P.cambridgei*, was not examined and therefore no taxonomical changes are proposed for this genus.

As a comparison, Fukushima and Bertani (2018) illustrated *Guyritacerrado* Guadanucci et al. 2007, both male and female characteristics. Primary and secondary copulation organs in both male and female specimens closely resemble those found in Psalmopoeinae, while the spermatheca morphology matches the one found in *P.elenae* comb. n. In male specimens, it features the megaspine PL on tibia of leg I. (Fukushima et al. 2018: fig. 20; also see Figure 8b in this work) which is present in Psalmopoeinae. Furthermore, the abdominal pattern is almost identical to the younger stages of *Pseudoclamoris* gen. n. species, while fading away when reaching maturity (Figure 13), although remaining present in *G.cerrado*.

According to the results presented here, phylogenetic relationship corresponds with the shape of both tibial apophysis and embolus. Aviculariinae genera form a clade with slender and strongly procurved embolus, as well as a lack of tibial apophysis while Psalmopoeinae genera are the sister group to the ground-dwelling Schismatothelinae with which they share similar structures, such as palpal bulb with long embolus bearing no keels and two tibial apophyses distally on the leg I. This grouping supports the hypothesis by Ortiz and Francke (2017) stating that “general shape of the pedipalpal bulb, types and position of the keels on the bulb, size of the tibia I accessory apophysis and spermatheca shape” are primary features for species groups and usable for species delimitation.

Morphological and DNA-based studies, including neotropical Ischnocolinae, are urgently needed to resolve the relationship of these taxa, as they are possibly more closely related than previously thought. It is necessary to find new morphological characteristics and, combined with molecular, geographic and ecological data, to undertake a more extensive and integrative analysis of Psalmopoeinae and possibly related taxa, as mentioned above (Hüsser et al. in prep).

Even though this contribution is a crucial step to better understand the diversity of Psalmopoeinae and their close relatives, information remains incomplete due to gaps in sampling, both molecular and morphological. The erection of the new genus *Pseudoclamoris* to include former *Tapinauchenius* species remains stable in both molecular and morphological analyses and thus lead to a better understanding of evolution of certain morphological characters, including stridulatory setae.

### Taxonomy

#### Araneae Clerck, 1757

##### Mygalomorphae Pocock, 1892

###### Theraphosidae Thorell, 1869

####### Psalmopoeinae

Taxon classificationAnimaliaAraneaeTheraphosidae

Samm & Schmidt, 2010

######## Included genera.

*Ephebopus*, *Psalmopoeus*, *Pseudoclamoris*, *Tapinauchenius*

######## Diagnosis.

The subfamily Psalmopoeinae is diagnosed by following synapomorphies as defined by Samm and Schmidt (2010), altered to fit the results presented here:

Psalmopoeinae can be distinguished from other new world subfamilies (save Aviculariinae) by their scopulae on anterior tarsi and metatarsi being extended laterally, giving a spatulate appearance and the absence of leg spines on tibiae and metatarsi. They differ from Aviculariinae (except females of *Pachistopelma* and *Iridopelmamarcoi* Bertani, 2012), as well as Theraphosinae by the lack of urticating setae on the opisthosoma. Females can further be distinguished from Schismatothelinae by their completely separated spermathecae. Males can easily be distinguished from those of Aviculariinae by the presence of two tibial apophyses on leg I.

######## Description.

Legs aspinose or with few apical spines on ventral tibiae and metatarsi; metatarsi and tarsi with scopulae very extended laterally, mainly on anterior legs, giving a spatulate appearance. Spermathecae consisting of two completely separated stalks. Male palpal bulb with long embolus without keels. Males with two tibial apophyses distally on the leg I. Type V urticating setae on prolateral palpal femur present (*Ephebopus*), or absent (all others). Stridulatory organ present (*Psalmopoeus*, *Pseudoclamoris*) or absent (all others). Legs weakly spined or aspinose, tarsi as broad as, or broader than metatarsi. Arboreal (*Psalmopoeus*, *Pseudoclamoris, Tapinauchenius*) and fossorial (*Ephebopus*, fossorial only as adults) species.

######## Distribution.

Mexico, Central America, north of South America and the Caribbean Islands.

**Figure 1.**
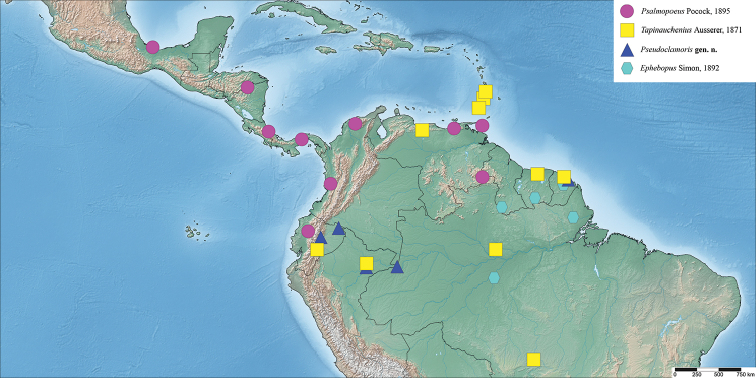
Distribution map of Psalmopoeinae.

**Figure 2.**
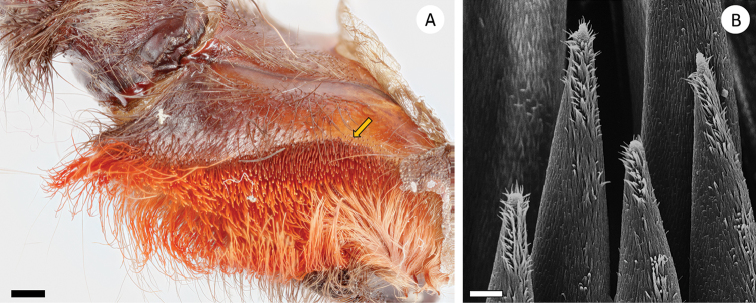
Field of needle-like setae on the proximal maxilla of *Pseudoclamoris* gen. n. **A** overview of the structure **B** detailed structure. Scale bar: 10 µm.

**Figure 3.**
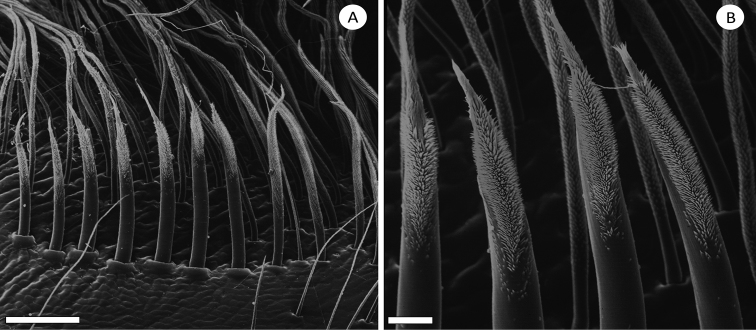
Lyra, proximal maxilla of *Psalmopoeus***A** overview of the structure **B** detailed structure. Scale bar: 10 µm.

####### Key to Psalmopoeinae genera

**Table:** 

1	Prolateral palpal femora with field of urticating setae	*** Ephebopus ***
–	Urticating setae absent	**2**
2	Prolateral maxillae without lyra	*** Tapinauchenius ***
–	Prolateral maxillae with lyra	**3**
3	Lyra consisting of small field of needle-shaped bristles	***Pseudoclamoris* gen. n.**
–	Lyra oval, consisting of short-shafted paddles; sometimes with distal blades	*** Psalmopoeus ***

##### Araneae Clerck, 1757

###### Mygalomorphae Pocock, 1892

####### Theraphosidae Thorell, 1869

######## Psalmopoeinae Samm & Schmidt, 2010

######### Pseudoclamoris gen. n.

Taxon classificationAnimaliaAraneaeTheraphosidae

http://zoobank.org/653225B7-2454-4F04-BD70-F7CBBAE7AD29

**Figure 4.**
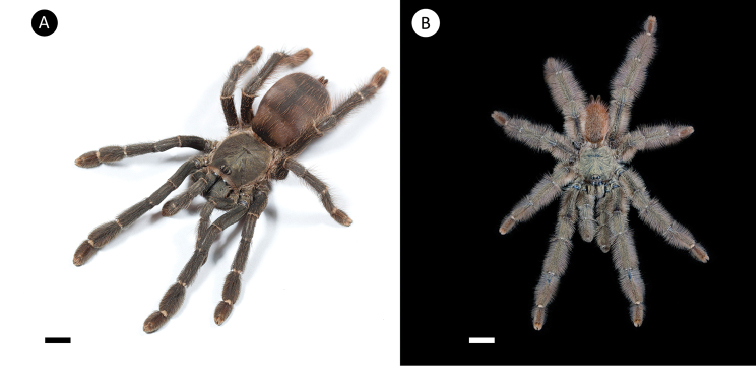
*Pseudoclamorisburgessi* sp. n. (habitus) **A** female specimen **B** male specimen. Scale bar: 5mm. Figure 5. *Pseudoclamorisburgessi* sp. n., male holotype – tibial apophyses **A** ventral **B** retrolateral **C** prolateral. Scale bar: 5mm.

**Figure 5.**
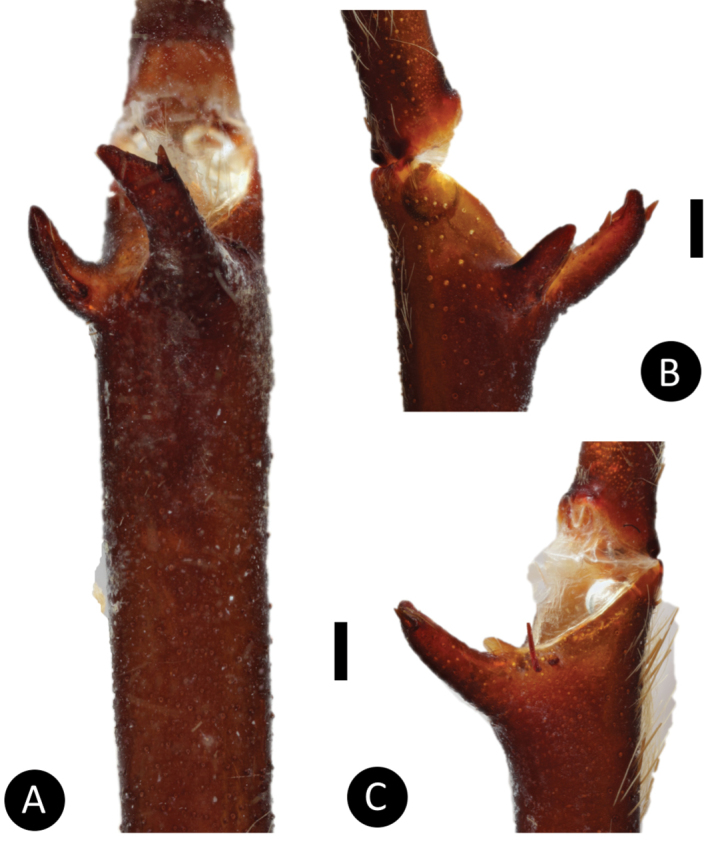
*Pseudoclamorisburgessi* sp. n., male holotype – tibial apophyses **A** ventral **B** retrolateral **C** prolateral. Scale bar: 5mm.

**Figure 6.**
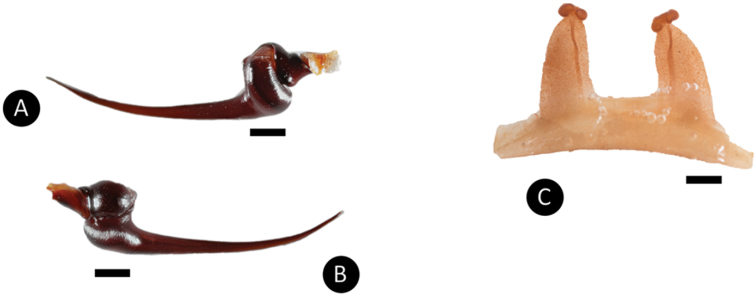
*Pseudoclamorisburgessi* sp. n., male holotype and female paratype **A** retrolateral view of palpal bulb **B** prolateral view of palpal bulb **C** spermathecae dorsal view. Scale bar: 5mm.

Figs 4 , 5 , 6 , 13 , 15b

########## Type species.

*Tapinaucheniusgigas* Caporiacco, 1954, herein designated.

########## Species included.

*Pseudoclamorisgigas* (Caporiacco, 1954), comb. n., *Pseudoclamoriselenae* (Schmidt, 1994), comb. n., and *Pseudoclamorisburgessi* sp. n.

########## Etymology.

The genus name derives from the Greek *pseudo* meaning false and the Greek *clamoris* meaning screaming/shouting translating into a “false screaming” group of Theraphosidae with the herein described feature of needle-like setae on the proximal maxilla, since the behaviour of *Pseudoclamoris* species resembles the one of *Psalmopoeus*, but no sound is audible when the specimens stridulate in defence posture. Gender is masculine.

########## Diagnosis.

Species of *Pseudoclamoris* differ from all known Psalmopoeinae, save *Psalmopoeus*, by the presence of a stridulatory organ on prolateral maxillae. They differ from *Psalmopoeus* by the specific shape of the stridulatory organ consisting of a field of needle-like bristles on the proximal maxilla (Figure 2) (vs. maxillary lyra oval in form, covering 1/4^th^ of surface, consisting of short shafted paddles with/without distal blades as in *Psalmopoeus*, compare with Mendoza 2014: figs. 3, 12–17). They furthermore differ from *Ephebopus* by the absence of urticating setae on palpal femora. Juveniles of *Pseudoclamoris* can be distinguished from those of *Tapinauchenius* by their ontogenetic colour change (Figure 15) and further in the first two instars by their distinct black herringbone pattern on a bright, slightly orange coloured opisthosoma in combination with a dark/black coloured metatarsus, while other leg segments are bright/brown coloured.

########## Description.

Carapace longer than wide with cephalic region slightly raised and convex. Striae well marked, fovea deep and straight. Chelicerae without rastellum. Eye tubercle distinct and wider than long, anterior eye row is (slightly) procurved and clypeus absent. Labium subquadrate, slightly wider than long, with numerous (100–300) cuspules concentrated on the anterior half. Maxillae subrectangular with anterior lobe distinctly produced into a conical form, with the inner angle bearing numerous cuspules (more than 100). Sternum longer than wide with posterior sigillae submarginal. STC with median row of a few small teeth. All leg tarsi and anterior metatarsi fully scopulated, Mt III scopulated at distal half and Mt IV scopulated only at distal third. Scopulae on anterior tarsi and metatarsi extended laterally resulting in a spatulate appearance. Femur IV without retrolateral scopula.

######### Pseudoclamoris burgessi sp. n.

Taxon classificationAnimaliaAraneaeTheraphosidae

http://zoobank.org/59A4D644-6FFC-493F-81EE-0881CD798AD7

Figs 4 , 5 , 6

########## Material examined.

Male holotype and female paratype from Leticia, Colombia deposited in SMF, leg. Auer, 2010; examined.

########## Other material examined.

1 female (MHCOL_00182) and 1 male (MHCOL_00201) from Iquitos, Peru in collection, leg. Auer; examined

########## Etymology.

The specific epithet is a noun in apposition as a recognition to Joseph Burges, USA who collected important material for this study.

########## Diagnosis.

Females of *Pseudoclamorisburgessi* differ from all other species of the genus by the lack of reddish setae on legs and opisthosoma (compare Figure 4a with Figure 13). From *P.gigas* comb. n. by the type locality and the shape of spermatheca where dual lobes appear to be more apart (compare with Schmidt 2003: figs. 6c, 614). Females differ from *P.elenae* comb. n. by the shape of spermatheca (dual lobed instead of multilobed as seen in *P.elenae* comb. n., Fig. 1 in Schmidt 1994b). Males of *P.burgessi* differ from those of other *Pseudoclamoris* species by the presence of an additional spine located distally on Rap.

########## Description of male holotype.

*Specimen preparation and condition*: The specimens (raised in captivity) were offspring of a wild caught specimen that were originally collected from a burrow and preserved in 80% ethanol. The original colouration of the caught specimen is faded due to the preservation. Right legs I, III, IV, and right pedipalp were removed for measurements and photographs and stored in vial with specimen. Tissue for DNA was extracted.

*General colouration*: The specimen is faded black/brown in colour *Cephalothorax*: Carapace 6.541 mm long and 5.214 mm wide; densely clothed with faded pubescence; appressed to the surface; fringe covered in long setae not closely appressed to the surface; hirsute appearance; foveal groove is medium deep and straight. The pars cephalica region rises very gradually from the foveal groove on a straight plane towards the ocular area; AER is procurved; PER is recurved; clypeus extends slightly on a curve. LBl 1.512 and LBw 2.891; sternum hirsute, clothed with faded brown, densely packed, short setae *Abdomen*: Densely clothed in short black/brown pubescence with numerous longer, lighter setae interspersed (generally red or orange *in situ*)

*Legs*: Hirsute; densely clothed in faded brown pubescence. Metatarsus I is straight. F1 14.123; F1w 3.142; P1 6.142; T1 11.521; M1 10.212; A1 6.125; F3 12.012; F3w 3.042; P3 4.062; T3 7.524; M3 8.145; A3 5.214; F4 13.012; F4w 3.042; P4 5.012; T4 11.102; M4 12.051; A4 5.105; femur III is normal. All tarsi are fully scopulate. Extent of the metatarsal scopulation, leg III (SC3) = 55% and leg IV (SC4) = 78%. Two ventral spinose setae are on metatarsus III, five ventral spinose setae on metatarsus IV, one prolateral spinose seta on tibia I, and one megaspine on the apex on the retrolateral branch of the tibial apophyses. *Coxa I*: Prolateral surface is covered by fine, hair-like setae. *Tibia I*: two apophyses that do not originate from a common base; Pap short and strong, with one short spine on inner face; the Rap is well developed, broad at its base with one short and strong spine on the inner face and two short and strong spines distally. *Pedipalps*: Hirsute, densely clothed in the same setal colour as the other legs, with numerous longer ventral setae, one spinose seta at the apical, prolateral femur and three prolateral spinose setae on the palpal tibia; PTl 7.012, PTw 2.125. When extended, embolus tapers with a curve to the retrolateral side; embolus slender, no keels; distinct dorsal and ventral transition from bulb to embolus

########## Description of female paratype.

*Specimen preparation and condition*: The examined specimens, raised in captivity, are offspring of wild caught specimen that were collected live from a burrow, and preserved in 80% ethanol. The original colouration has faded due to the preservation. A 50 mg tissue sample was extracted for DNA analysis. The genital plate with spermathecae was removed and cleared, then stored in a vial with the specimen.

*General colouration*: faded black/brown. *Cephalothorax*: Carapace is 17.456 mm long, and 16.245 mm wide; densely clothed with short faded black/brown pubescence, closely appressed to the surface, the fringe densely covered in slightly longer setae; foveal groove is medium deep and slightly procurved. The pars cephalica region gently rises from the thoracic furrow, arching anteriorly toward the ocular area; AER is slightly procurved, PER is very slightly recurved; clypeus extends forward on a curve. LBl 1.627 and LBw 2.982; sternum is covered with short faded setae. The abdomen is densely clothed dorsally in short faded black setae with longer, lighter setae (generally red or orange in situ). *Spermathecae*: paired and separate, with capitate bulbs widening towards the bases and not fused, two lobes on each end. *Legs*: are densely clothed in short faded black/brown pubescence. F1 16.145; F1w 4.234; P1 8.241; T1 13.356; M1 11.278; A1 7.252; F3 11.412; F3w 3.845; P3 6.265; T3 9.156; M3 11.589; A3 6.256; F4 14.163; F4w 4.167; P4 7.532; T4 12.356; M4 13.578; A4 6.521. All tarsi are fully scopulate. Extension of metatarsal scopulation: Leg III (SC3) = 58% and leg IV (SC4) = 79%. There is one ventral and one prolateral spinose seta on metatarsus III, and four ventral spinose setae and one prolateral spinose seta on metatarsus IV. *Coxa I*: prolateral surface is covered by very thin tapered and fine, hair-like setae. *Pedipalps*: densely clothed in the same setal colour as the other legs, with one spinose seta on the apical, prolateral femur, four prolateral (two at the apical, prolateral border with the tarsus) spinose setae and one ventral spinose seta on the tibia.

########## Distribution and natural history.

Lowland rainforest of the Amazon region of the countries of Colombia and Peru. Ecuador as distribution is highly likely due to the occurrence very close to the border of Ecuador in Leticia, Columbia. *Pseudoclamorisburgessi* is sympatric with *Tapinauchenius* sp. in the same mentioned region (Auer, pers. comm.)

########## Remarks.

No threat through poaching or smuggling of animals out of the country of origin is to be expected, since *P.burgessi* is bred in the pet-trade all around the world for several years now. Egg sacs of *P.burgessi* typically contain between 80 and 220 spiderlings, depending on the size of the female. (pers. obs. and Rast pers. comm.)

##### Araneae Clerck, 1757

###### Mygalomorphae Pocock, 1892

####### Theraphosidae Thorell, 1869

######## Psalmopoeinae Samm & Schmidt, 2010

######### Tapinauchenius

Taxon classificationAnimaliaAraneaeTheraphosidae

Ausserer, 1871

########## Type species.

*Tapinaucheniusplumipes* (C. L. Koch, 1842).

########## Species included.

*Tapinaucheniusbrunneus* Schmidt, 1995, *Tapinaucheniuscupreus* Schmidt & Bauer, 1996, *Tapinaucheniuslatipes* L. Koch, 1875, *Tapinaucheniussanctivincenti* (Walckenaer, 1837), *Tapinaucheniusviolaceus* (Mello-Leitão, 1930), *Tapinaucheniusrasti* sp. n., *Tapinaucheniuspolybotes* sp. n.

########## Diagnosis.

Differs from all other Psalmopoeinae genera by the lack of a stridulatory organ palpal coxa, prolateral and from *Ephebopus* by the absence of urticating setae on palpal femora.

########## Description.

Carapace longer than wide with cephalic region slightly raised and convex. Striae well marked, fovea deep and straight. Chelicerae without rastellum. Eye tubercle distinct and wider than long, anterior eye row is straight and clypeus absent. Labium subquadrate, slightly wider than long, with numerous (100–300) cuspules concentrated on the anterior half. Maxillae subrectangular with anterior lobe distinctly produced into a conical form, with the inner angle bearing numerous cuspules (more than 100). Sternum longer than wide with posterior sigillae submarginal. STC with median row of a few small teeth. All leg tarsi and anterior metatarsi fully scopulated, Mt III scopulated at distal half and Mt IV scopulated only at distal third. Scopulae on anterior tarsi and metatarsi extended laterally resulting in a spatulate appearance. Femur IV without retrolateral scopula.

########## Remarks.

Species of the genus *Tapinauchenius* do not show any ontogenetic pattern change. Juveniles are uniformly coloured in a dark-grey with faint bluish tone (Figure 15a).

######### Tapinauchenius polybotes sp. n.

Taxon classificationAnimaliaAraneaeTheraphosidae

http://zoobank.org/12D484B9-3C64-4E80-8251-E2C41F91C90E

Figs 7 , 8 , 9

########## Material examined.

Male holotype and female paratype from Saint Lucia, Lesser Antilles deposited in SMF, leg. Sanchez, don. B Rast, 2013; examined.

########## Other material examined.

1 female (MHCOL_0034) and 1 male (MHCOL_0048)

########## Etymology.

The specific epithet is a noun in apposition, referring to the giant Polybotes originating in the Greek mythology and is in reference to the large size of the species compared to congeners of the genus and subfamily in general.

########## Diagnosis.

*Tapinaucheniuspolybotes* differs from all other *Tapinauchenius* species by its large overall size in both male and female specimens and the fact that it’s only known from the type locality, with high possibility of endemism due to its location.

Males additionally differ from all other *Tapinauchenius* species by having the embolus strongly S-curved to retrolateral side in apical fourth (Figure 9c), otherwise only known from species of *Ephebopus*, as shown by West et al. (2008: Figs 7–9).

########## Description of male holotype.

*Specimen preparation and condition*: Adult female collected at the type locality in 2010 by A Sanchez. In captivity, female built an egg sack of which the specimens (holotype and paratype) were raised to adulthood and then donated to the author by B Rast, collected alive as adult specimens, preserved in 80% ethanol; original colouration faded due to preservation. Right legs I, III, IV, and right pedipalp removed for measurements and photographs; stored in vial with specimen. Tissue for DNA was extracted.

*General colouration*: Faded black/blueish. *Cephalothorax*: Carapace 19.421 mm long, 16.412 mm wide; densely clothed with faded pubescence, appressed to surface; fringe covered in long white setae not closely appressed to surface, hirsute appearance; foveal groove medium deep and straight; pars cephalica region rises very gradually from foveal groove on a straight plane towards the ocular area; AER procurved, PER recurved; clypeus extends slightly on a curve; LBl 3.314, LBw 3.021; sternum hirsute, clothed with faded, densely packed, short setae. *Abdomen*: Densely clothed in short black/brown pubescence with numerous longer, lighter setae . *Legs*: Hirsute; densely clothed in faded pubescence. Metatarsus I straight. F1 16.962; F1w 4.232; P1 9.171; T1 14.816; M1 12.983; A1 7.151; F3 13.892; F3w 3.895; P3 7.462; T3 11.122; M3 9.834; A3 7.167; F4 17.916; F4w 4.641; P4 8.266; T4 13.873; M4 16.368; A4 8.143; femur III is normal. All tarsi fully scopulate. Extent of metatarsal scopulation: leg III (SC3) = 60%; leg IV (SC4) = 78%. Two ventral spinose setae on metatarsus III; five ventral spinose setae on metatarsus IV; one prolateral spinose seta on tibia I; one megaspine on the apex on the retrolateral branch of the tibial apophyses. *Coxa I*: Prolateral surface covered by fine, hair-like setae. *Tibia I*: two apophyses that do not originate from a common base, Pap short and strong, with one short spine on inner face; the Rap is well developed, broad at its base with one short and strong spine on the inner face and two short and strong spines on top; *Pedipalps*: Hirsute; densely clothed in the same setal colour as the other legs, with numerous longer ventral setae; one spinose seta at the apical, prolateral femur and three prolateral spinose setae on the palpal tibia. PTl 7.145, PTw 2.145. Palpal bulb large, globular, short slender embolus tapering slightly apically, two times longer than the tegulum, “S” shapely curved to retrolateral side on apical fourth (Figure 9c). The embolus base shows a clear separation from the tegulum, with the width of the embolus base 3/5 of the tegulum height.

########## Description of female paratype.

*Specimen preparation and condition*: Origin same as holotype; collected alive, preserved in 80% ethanol. The original colouration has faded due to the preservation. A 50 mg tissue sample was extracted for DNA analysis.

*General colouration*: faded black. *Cephalothorax*: carapace is 20.14 mm long and 16.542 mm wide. It is densely clothed with short faded black/brown pubescence closely appressed to surface, the fringe is densely covered in slightly longer setae; foveal groove is medium deep and slightly procurved; cephalic region gently rises from the thoracic furrow, arching anteriorly toward the ocular area. AER is slightly procurved; PER very slightly recurved; clypeus extends forward on a curve; LBl 3.514, LBw 3.014. The sternum is covered with short faded setae. *Abdomen*: densely clothed dorsally in short faded black setae with longer, lighter setae (generally red *in situ*). *Spermathecae*: paired and separate, with capitate bulbs widening towards the bases and not fused. *Legs*: densely clothed in short faded black/blue pubescence. F1 17.132; F1w 5.142; P1 9.214; T1 15.212; M1 13.213; A1 7.211; F3 14.212; F3w 4.215; P3 7.512; T3 11.32; M3 10.132; A3 7.321; F4 18.217; F4w 5.241; P4 8.576; T4 14.576; M4 16.678; A4 8.213. All tarsi are fully scopulate. Extent of the metatarsal scopulation: leg III (SC3) = 66% and leg IV (SC4) = 54%. Ventral and prolateral spinose setae on metatarsus IV, five ventral spinose setae and one prolateral spinose seta on metatarsus IV. *Pedipalps*: densely clothed in the same setal colour as the other legs.

########## Distribution and natural history.

Only known from the island of Saint Lucia, Lesser Antilles.

########## Remarks.

In pet trade, specimens labelled as *Tapinaucheniussanctivincenti* and Tapinaucheniuscf.sanctivincenti belong to the same species described here as *Tapinaucheniuspolybotes* sp. n. Pet trade material originates from the mother of the type material of the species herein described. Shortly after introduction to the hobby, the species was available under the name of Tapinaucheniuscf.sanctivincenti and *Tapinauchenius* sp. “St. Lucia”.

No threat through poaching or smuggling of animals out of the country of origin is to be expected, since *T.polybotes* is breed successfully in the pet trade all around the world since 2012. Egg sac of *T.polybotes* typically contain between 80 and 190 spiderlings, depending on the size of the female. (pers. obs. and B Rast pers. comm.)

**Figure 7.**
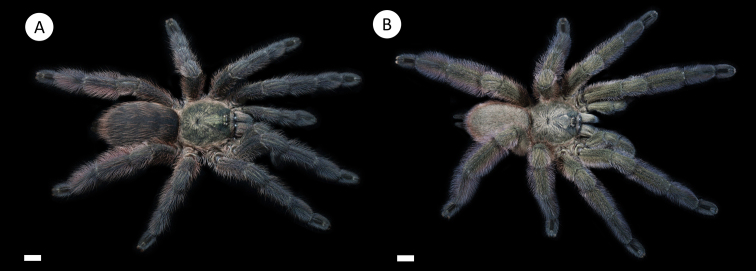
*Tapinaucheniuspolybotes* sp. n., (habitus) **A** female specimen, paratype **B** male specimen, holotype. Scale bar: 5mm.

**Figure 8.**
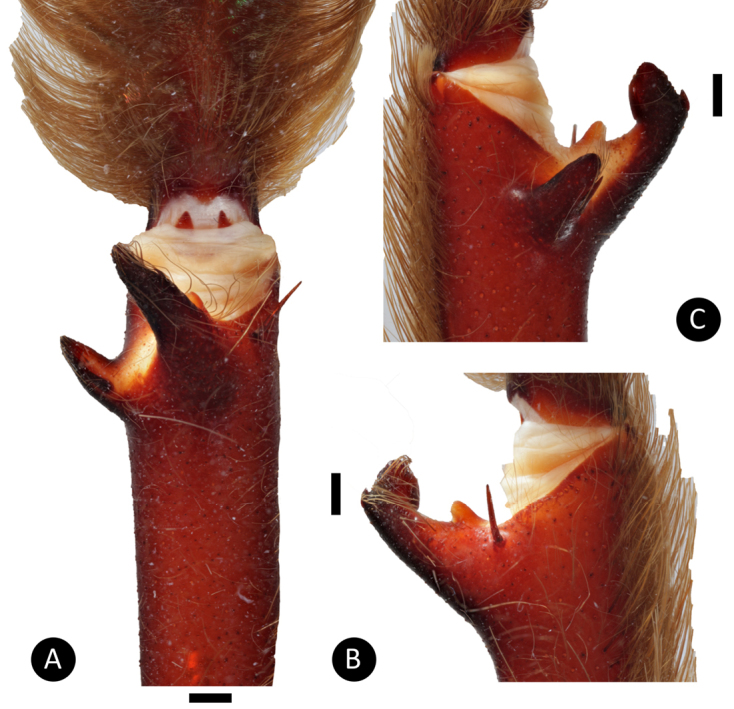
*Tapinaucheniuspolybotes* sp. n., male holotype – tibial apophyses **A** ventral **B** retrolateral **C** prolateral. Scale bar: 5mm.

**Figure 9.**
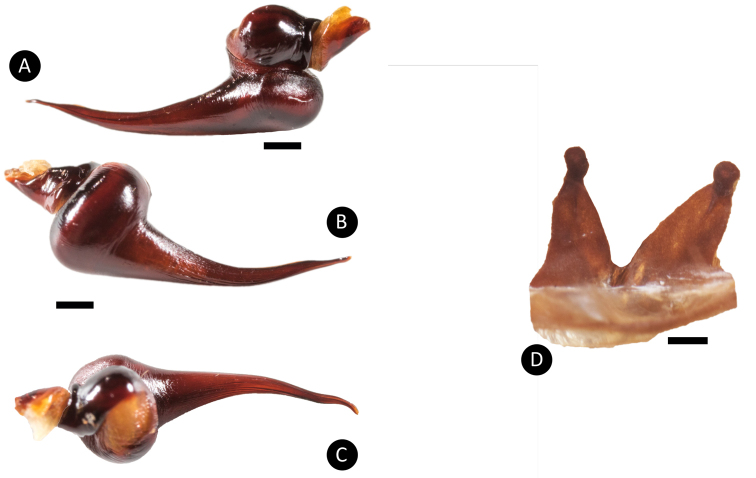
*Tapinaucheniuspolybotes* sp. n., male holotype and female paratype **A** retrolateral view of palpal bulb **B** prolateral view of palpal bulb **C** distal view of palpal bulb **D** spermathecae dorsal view. Scale bar: 5mm.

######### Tapinauchenius rasti sp. n.

Taxon classificationAnimaliaAraneaeTheraphosidae

http://zoobank.org/4261DE10-B337-43EB-A137-AE34FA7589CE

Figs 10 , 11 , 12

########## Material examined.

Male holotype and female paratype from region around Mt. Taboi, Union Island, St. Vincent and the Grenadines, Lesser Antilles deposited in SMF, leg. Burgess, don. B Rast, 2013; examined.

########## Other material examined.

1 female (MHCOL_0087) and 2 males (MHCOL_0076, 0065).

########## Etymology.

The specific epithet is a noun in apposition as a recognition to Bastian Rast, Switzerland, who guided the author in his early years of tarantula research and is still tremendously supportive of the author’s work.

########## Diagnosis.

**Figure 10.**
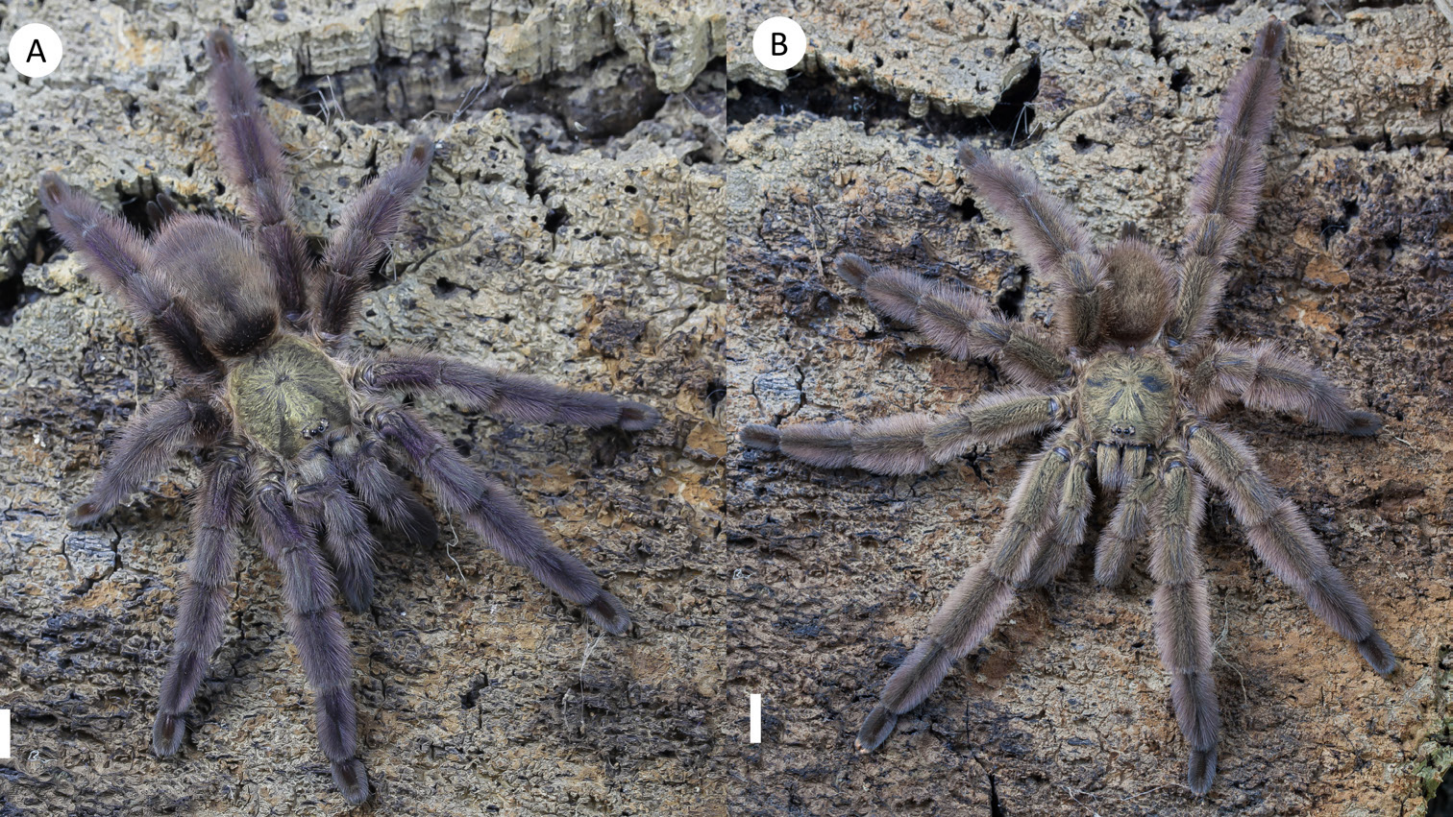
*Tapinaucheniusrasti* (habitus) **A** female specimen, paratype **B** male specimen, holotype. Scale bar: 5mm.

**Figure 11.**
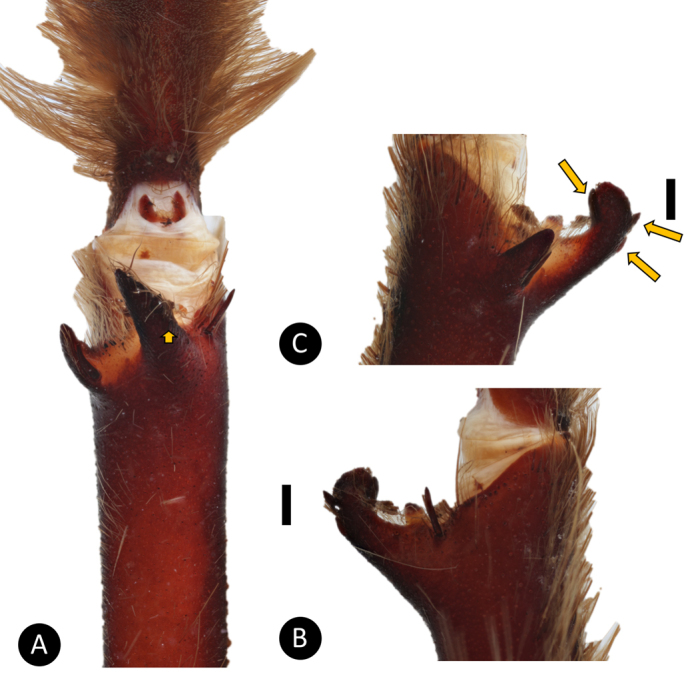
*Tapinaucheniusrasti*, male holotype – tibial apophyses **A** ventral **B** retrolateral **C** prolateral. Scale bar: 5mm.

*Tapinaucheniusrasti* sp. n. differs from all known *Tapinauchenius* by their type locality and unique colouration in adult females. Males furthermore differ from those of *T.polybotes* by their slenderer embolus and from all other *Tapinauchenius* species by having a Rap with three short and strong spines (Figure 11c). Females can be distinguished from those of all other *Tapinauchenius* by their adult colouration (see Figure 10), having a brightly green coloured cephalothorax while its abdomen and legs are of blue to violet colour with longer reddish setae on legs III and IV.

########## Description of male holotype.

*Specimen preparation and condition*: In captivity, female built an egg sack from which the specimens (holotype and paratype) were raised to adulthood and donated to the author by B Rast, collected alive, preserved in 80% ethanol. The original colouration has faded due to the preservation. A 50 mg tissue sample was extracted for DNA analysis. Right legs I, III, IV, and right pedipalp removed for measurements and photographs; stored in vial with specimen. Tissue for DNA was extracted.

*General colouration*: Faded black/blueish. *Cephalothorax*: Carapace 16.421 mm long, 14.412 mm wide; densely clothed with faded pubescence, appressed to surface; fringe covered in long white setae not closely appressed to surface, hirsute appearance; foveal groove medium deep and straight; pars cephalica region rises very gradually from foveal groove on a straight plane towards the ocular area; AER procurved, PER recurved; clypeus extends slightly on a curve; LBl 2.104, LBw 2.231; sternum hirsute, clothed with faded, densely packed, short setae. *Abdomen*: Densely clothed in short black/brown pubescence with numerous longer, lighter setae.

*Legs*: Hirsute; densely clothed in faded pubescence. Metatarsus I straight. F1 14.961; F1w 4.032; P1 5.714; T1 13.210; M1 11.973; A1 6.013; F3 10.753; F3w 3.832; P3 4.692; T3 9.072; M3 11.312; A3 5.173; F4 13.112; F4w 4.252; P4 5.342; T4 13.102; M4 13.552; A4 5.981; femur III is normal. All tarsi fully scopulate. Extent of metatarsal scopulation: leg III (SC3) = 59%; leg IV (SC4) = 72%. Two ventral spinose setae on metatarsus III; five ventral spinose setae on metatarsus IV; one prolateral spinose seta on tibia I; one megaspine on the apex on the retrolateral branch of the tibial apophyses. *Coxa I*: Prolateral surface covered by fine, hair-like setae. *Tibia I*: two apophyses that do not originate from a common base, Pap short and strong, with one short spine on inner face; the Rap is well developed, broad at its base with one short and strong spine on the inner face and three short and strong spines on top (see arrows Figure 11c); *Pedipalps*: Hirsute; densely clothed in the same setal colour as the other legs, with numerous longer ventral setae; one spinose seta at the apical, prolateral femur and three prolateral spinose setae on the palpal tibia. PTl 7.123, PTw 2.154. Palpal bulb large, globular, short slender embolus tapering slightly apically. When extended, the embolus tapers with a curve to the retrolateral side; no keels; distinct dorsal and ventral transition from bulb to embolus.

########## Description of female paratype.

*Specimen preparation and condition*: offspring of wild-caught specimen raised in captivity, specimen collected live from burrow50mg tissue sample extracted for DNA analysis. Genital plate with spermathecae removed and cleared, stored in vial with specimen.

*General colouration*: Faded black/brown. *Cephalothorax*: Carapace 16.553 mm long, 13.634 mm wide; metallic green colouration in live specimens, densely clothed with short faded black/brown pubescence closely appressed to surface; fringe densely covered in slightly longer setae; foveal groove medium deep and slightly procurved; pars cephalica region gently rises from thoracic furrow, arching anteriorly toward ocular area; AER procurved, PER very slightly recurved; clypeus extends forward on a curve; LBl 2.221, LBw 2.521; sternum hirsute, clothed with short faded setae. *Abdomen*: Densely clothed dorsally in short faded black setae with longer, lighter setae (generally red in situ). *Spermathecae*: Paired and separate, with capitate bulbs widening towards the bases; not fused.*Legs*: Hirsute; densely clothed in short faded blue pubescence; F1 14.432; F1w 4.012; P1 7.212; T1 11.413; M1 9.312; A1 5.512; F3 10.857; F3w 4.132; P3 6.142; T3 8.581; M3 9.731; A3 5.321; F4 13.214; F4w 4.235; P4 7.125; T4 11.235; M4 12.456; A4 6.236. All tarsi fully scopulate. Extent of metatarsal scopulation: leg III (SC3) = 58%; leg IV (SC4) = 74%.

One ventral and one prolateral spinose seta on metatarsus III; four ventral spinose setae and one prolateral spinose seta on metatarsus IV. *Coxa* I: Prolateral surface covered by very thin tapered and fine, hair-like setae. *Pedipalps*: Densely clothed in the same setal colour as the other legs; one spinose seta on the apical, prolateral femur, four prolateral (two at the apical, prolateral border with the tarsus) spinose setae and one ventral spinose seta on the tibia.

########## Distribution and natural history.

Only known from Union Island, Caribbean. All adult specimens were observed on larger/older growth trees and used cavities and “knot holes” as retreats. One subadult specimen observed under loose bark. Retreats were typically silk-lined around the entrance. Sub adults were seen on large and small diameter trees as well as bromeliads, rocks and one occurrence on the ground. Females showed parental care. Two different females observed with spiderlings in and around retreat entrance. (pers. comm. Joseph Burgess).

########## Remarks.

In the pet trade, specimens labelled as *Tapinauchenius* sp. “Caribbean Diamond” and *Tapinauchenius* sp. “Union Island” belong to the same species described here as *Tapinaucheniusrasti* sp. n.

No threat through poaching or smuggling of animals out of the country of origin is to be expected, since *T.rasti* sp. n. is bread successfully in the pet-trade all around the world since 2012. Egg sac of *T.rasti* sp. n. typically contain between 80 and 190 spiderlings, depending on the size of the female. (pers. obs. and Rast pers. comm.)

**Figure 12.**
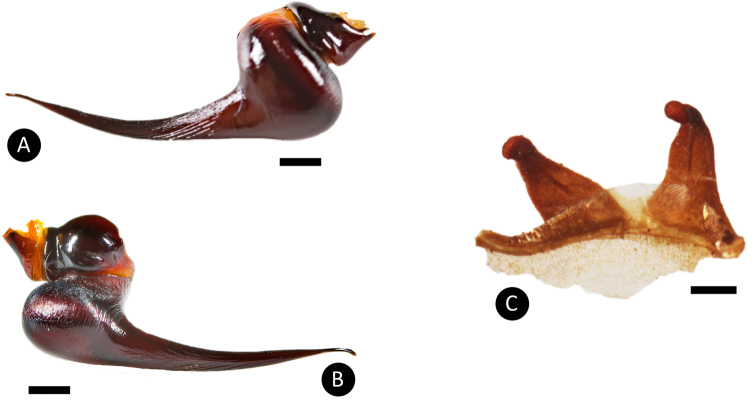
*Tapinaucheniusrasti* sp. n., male holotype and female paratype **A** retrolateral view of palpal bulb **B** prolateral view of palpal bulb **C** spermathecae dorsal view. Scale bar: 5mm.

**Figure 13.**
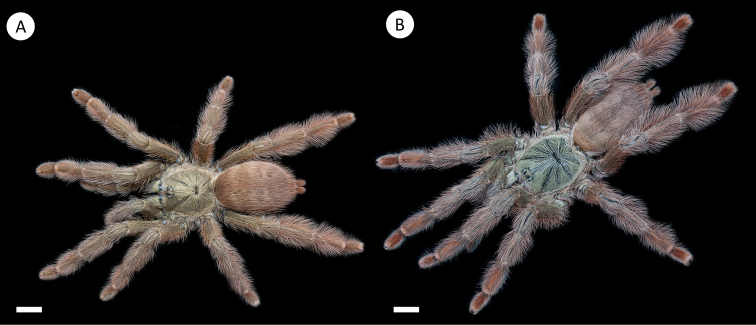
*Pseudoclamoris* spp. (habitus) **A** female of *Pseudoclamoriselenae* comb. n. **B** female specimen of *Pseudoclamorisgigas* comb. n. Scale bar: 5mm.

**Figure 14.**
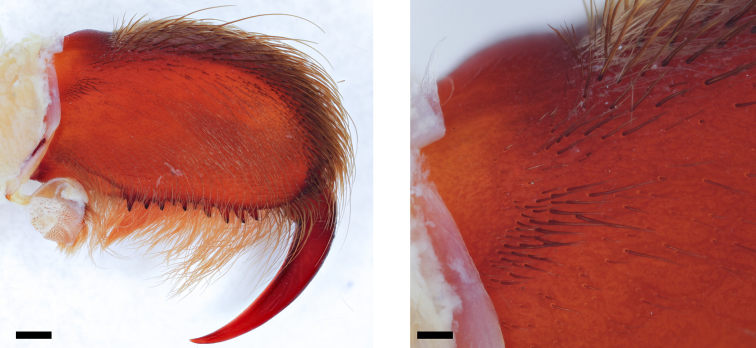
Cheliceral strikers of *Tapinaucheniusviolaceus***A** overview **B** detailed view. Scale bars: 20 µm (**A**); 100 µm (**B**).

**Figure 15.**
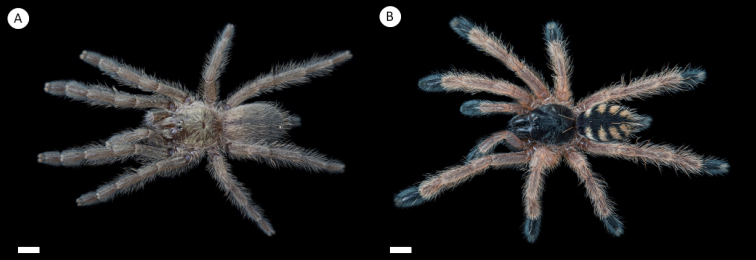
Ontogenetic pattern change **A** juvenile of *Tapinauchenius***B** juvenile of *Pseudoclamoris*. Scale bar: 5mm.

**Figure 16.**
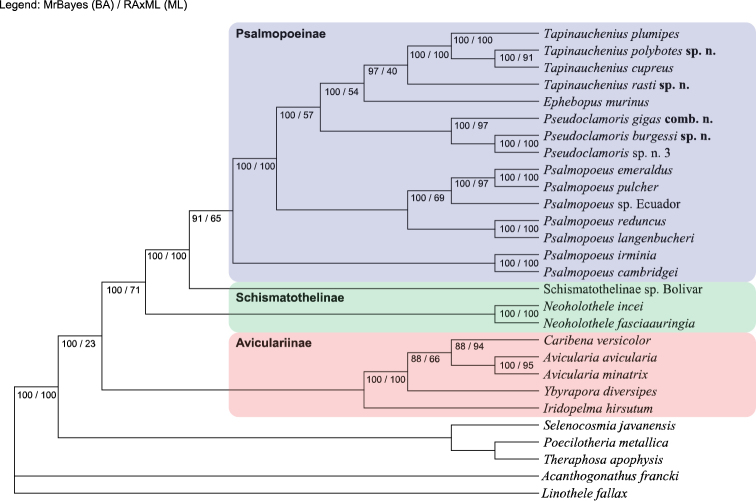
Phylogenetic tree of Psalmopoeinae species and close relatives, based upon combined CO1, 16S and 28S genomic region analysis, combined with ML and BA values according to defined parameters.

##### Species inquirenda

###### Tapinauchenius sanctivincenti

Taxon classificationAnimaliaAraneaeTheraphosidae

(Walckenaer, 1837)

Mygale sancti-vincentii Walckenaer, 1837: 216 (Df).
Tapinauchenius sanctivincenti Simon, 1892e: 553.
Tapinauchenius sanctivincenti FO Pickard-Cambridge, 1896: 745, pl. 34, f. 21.

####### Remarks.

The type material, presumably deposited in MNHN, could not be located in the museum collection and is considered lost by the curator (Rollard, pers. comm.). Due to the given type locality of St. Vincent Island it is possible to allocate new material in order to designate a neotype (in prep.).

##### Nomen dubium

###### Tapinauchenius subcaeruleus

Taxon classificationAnimaliaAraneaeTheraphosidae

Bauer & Antonelli, 1997

Tapinauchenius subcaeruleus Bauer & Antonelli, 1997: 429, f. 1–3 .

####### Remarks.

The original description by Bauer and Antonelli (1997) stated the authors intended to deposit a female holotype at SMF. However, this holotype could not be located in the collections and has never been deposited there (Jäger, pers. comm.). As there is no holotype and the description does not allow for a precise diagnosis, and as further characterisation of the species and the provided type locality (Ecuador without specification) are too broad, it is impossible to allocate new material. Consequently, the species is herein considered a *nomen dubium*.

**Figure 17.**
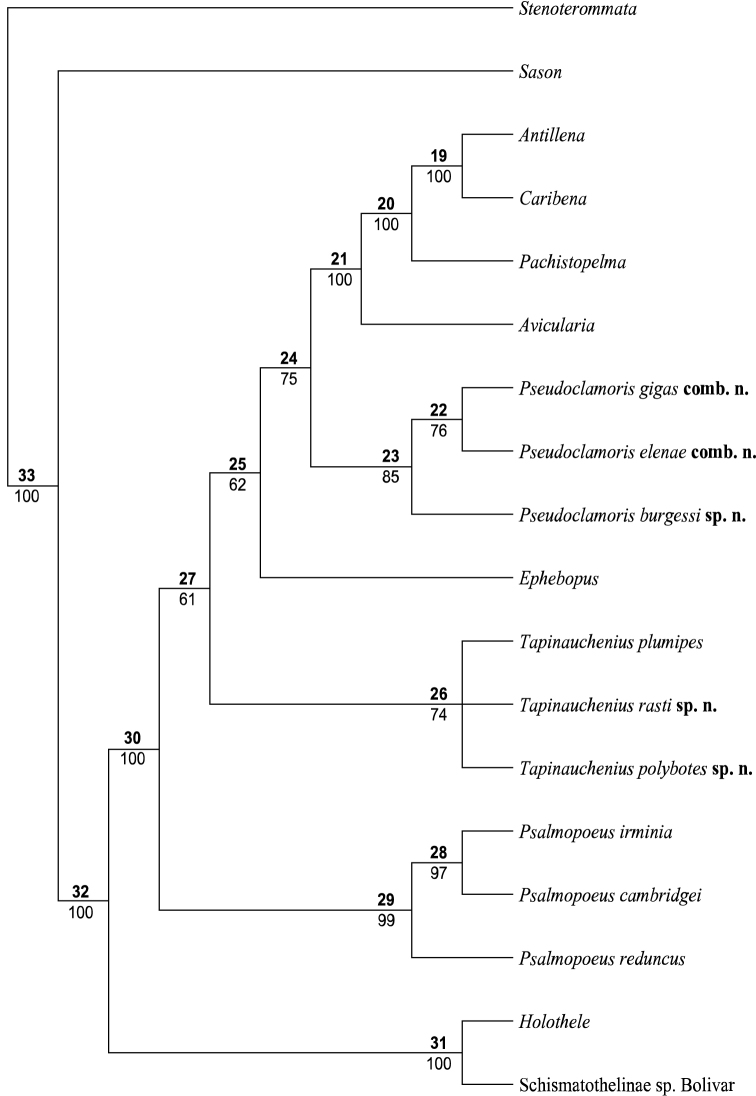
Single tree resulting from cladistic analysis (Length = 58, Ci = 0.7241, Ri = 0.8298)

## Supplementary Material

XML Treatment for Psalmopoeinae

XML Treatment for Pseudoclamoris

XML Treatment for Pseudoclamoris burgessi

XML Treatment for Tapinauchenius

XML Treatment for Tapinauchenius polybotes

XML Treatment for Tapinauchenius rasti

XML Treatment for Tapinauchenius sanctivincenti

XML Treatment for Tapinauchenius subcaeruleus

## Supplementary materials

Supplementary material 1 Dryad Data Info Data type: measurement This is an open access article distributed under the terms of the Creative Commons Attribution License (CC BY 4.0), which permits unrestricted use, distribution, and reproduction in any medium, provided the original author and source are credited.Martin Hüsser
